# 2170 Risk factors for prescription opioid misuse after traumatic injury in adolescents

**DOI:** 10.1017/cts.2018.302

**Published:** 2018-11-21

**Authors:** Teresa M. Bell, Christopher A. Harle, Dennis P. Watson, Aaron E. Carroll

## Abstract

OBJECTIVES/SPECIFIC AIMS: The objective of this study is to determine predictors and motives for sustained opioid use, prescription misuse, and nonmedical opioid use in the adolescent trauma population. METHODS/STUDY POPULATION: This is a prospective cohort study that will follow patients for 1 year and administer surveys to patients on prescription opioid usage; substance use; utilization of pain management and mental health services; mental and physical health conditions; and behavioral and social risk factors. Patient eligibility criteria include: (1) patient is 12–18 years of age; (2) admitted for trauma; (3) english speaking; (4) resides within Indianapolis, IN metropolitan area; and (5) consent can be obtained from a parent or guardian. Patients with severe brain injuries or other injuries that prevent survey participation will be excluded. The patient sample will comprise of 50 traumatically injured adolescents admitted for trauma who will be followed for 12 months after discharge. RESULTS/ANTICIPATED RESULTS: We expect that the results of this study will identify multiple risk factors for sustained opioid use that can be used to create targeted interventions to reduce opioid misuse in the adolescent trauma population. Clinical predictors such as opioid type, dosage, and duration that can be modified to reduce the risk of long-term opioid use will be identified. We expect to elucidate clinical, behavioral, and social risk factors that increase the likelihood adolescents will misuse their medication and initiate nonmedical opioid use. DISCUSSION/SIGNIFICANCE OF IMPACT: Trauma is a surgical specialty that often has limited collaboration with behavioral health providers. Collaborative care models for trauma patients to adequately address the psychological impact of a traumatic injury have become more common in recent years. These models have primarily been concerned with the prevention of post-traumatic stress disorder. We would like to apply the findings of our research to better understand what motivates adolescents to misuse pain medications as well as how clinical, individual, behavioral, and social factors affect medication usage. This may help identify patients at greater risk of developing a SUD by asking questions not commonly addressed in the hospital setting. For example, similar to how trauma centers have mandated brief interventions on alcohol use be performed for center verification, screening patients’ on their social environment may identify patients at greater risk for SUD than assumed. The long-term goal would be to prevent opioid use disorders in injured adolescents by providing better post-acute care support, possibly by developing and implementing a collaborative care model that addresses opioid use. Additionally, we believe our findings could be applied in the acute care setting as well to help inform opioid prescribing and pain management methods in the acute phase of an injury. Genetic testing to determine which opioid to prescribe pediatric surgical patients is starting to be done at some pediatric hospitals. Certain genes determine which specific opioid is most effective in controlling a patient’s pain and, further, using the optimal opioid medication can also reduce overdose. Our findings may help refine prescribing patterns that could increase or decrease the likelihood of developing SUD in patients with certain genetic, clinical, behavioral, and social characteristics.

